# Association of infertility treatments and incidence of stroke among women: a systematic review

**DOI:** 10.1530/RAF-24-0120

**Published:** 2025-10-31

**Authors:** Asha Vijay, Vaishali Dagar, Rani Priyanka, Anitha Anuganti Manoj, Murali Mohan Reddy Gopireddy, Ajay Krishan Adusumilli

**Affiliations:** ^1^ Department of Reproductive Medicine, Garbhagudi IVF Centre, Bangalore, Karnataka, India; ^2^ Department of Evidence Synthesis, coGuide Academy, Bangalore, Karnataka, India

**Keywords:** cerebrovascular, infertility, stroke, women

## Abstract

**Abstract:**

Infertility affects a significant proportion of the global population, leading to an increased demand for infertility treatments among women. There are concerns about the potential association between infertility treatment and stroke. However, this potential association remains poorly understood. The study aimed to address this evidence gap by comprehensively examining the available evidence on the incidence of stroke among women who underwent infertility treatments. The study included retrospective and prospective cohort studies on women who had a history of infertility treatments and were assessed for cerebrovascular accident or stroke. The primary outcome was the incidence of any type of stroke. We searched PubMed, Embase, Cochrane, Web of Science and CINAHL databases using a structured search strategy. Out of 1,076 identified studies, six were included in qualitative synthesis, with a pooled sample size of 406,438 women. Meta-analysis could not be performed due to significant heterogeneity across the studies. The reported incidence of any stroke varied from 16 to 437 per 100,000 person-years. The associations between ART treatments and stroke were reported with HR ranging from 0.82 (0.68–0.99) to 1.66 (1.17–2.35). The evidence on the association between infertility treatment and stroke is still emerging, hampering definitive conclusions regarding the strength of associations due to the limited number of studies. Key questions remain unanswered regarding postpartum stroke, overall stroke incidence among infertile women, and the specific factors contributing to stroke risk. Future research should focus on prospective studies to elucidate these associations, guiding clinical practice and patient care effectively.

**Lay summary:**

Infertility affects many people worldwide, leading more women to seek medical treatments to help them conceive. As these treatments become more common, there have been questions about whether they might increase the risk of stroke. We reviewed all available research studies that looked at stroke occurrence in women who had received infertility treatments. After searching through medical research databases, we found six relevant studies that together included over 400,000 women. On observation of 100,000 women per year, the number of stroke cases showed wide variation, ranging from as low as 16 to as high as 437. Some studies suggest that fertility treatments (ART) may slightly raise the risk of stroke, while one study found a small decrease in risk. However, these studies were quite different from each other in terms of who they studied and how they measured outcomes, making it difficult to draw firm conclusions. Important questions still need to be answered, such as whether infertile women have a higher stroke risk overall, whether there is an increased risk right after giving birth, and what specific factors might contribute to stroke risk. More research, particularly studies that follow women over time, is needed to better understand these risks and help doctors provide the best care for their patients.

## Introduction

Infertility is defined as the inability to achieve pregnancy after 12 months of unprotected intercourse. It affects approximately one in six people globally (World Health Organization 2023, 2024). According to the Global Burden of Disease (GBD) data, approximately 55 million men and 110 million women were affected by infertility in 2021. Between 1990 and 2021, infertility rates have shown a steady upward trend, with women experiencing infertility at nearly twice the rate of men (Feng *et al.* 2025). This growing global burden of infertility has significantly increased the demand for infertility treatments.

Parallelly, there have been significant advancements in assisted reproductive technologies (ART), which encompass various interventions from medications to ART (NICHD – Eunice Kennedy Shriver National Institute of Child Health & Human Development 2017). Advancements in accessibility and affordability have led to a rapid rise in the utilization of these treatments (Gleicher 2018). The International Committee for Monitoring Assisted Reproductive Technologies (ICMART) reported that in 2018 alone, around 3.56 million ART cycles were conducted, resulting in an estimated 870,000 births (Chambers *et al.* 2021). Furthermore, a recent global estimate spanning 1978–2018 indicated that between 9.3 million and 12.4 million babies were born through ART across 101 countries – highlighting the exponential growth in ART utilization over the past four decades (Adamson *et al.* 2025).

Despite the proven safety of ART interventions, concerns regarding potential adverse effects and medical complications persist. A significant concern associated with infertility treatments is the potential long-term risk of cardiovascular diseases, particularly stroke among women (Dayan *et al.* 2017, Magnus *et al.* 2023). There is a considerable body of evidence regarding the predisposition of pregnant women to various cardiovascular complications, such as venous thromboembolism, coronary ischemia, heart failure, and stroke (Mehta *et al.* 2015). Other medications, such as oral contraceptives and hormone therapy, have also been proven to increase the risk of cardiovascular conditions among women of reproductive age group (Henderson & Lobo 2012, Ge *et al.* 2019).

The relationship between infertility treatments and stroke remains inadequately understood. There have been sporadic reports and few case studies linking infertility treatments with stroke. There have been few attempts recently to explore this association, with conflicting findings. A comprehensive evidence synthesis effort to consolidate the body of existing evidence is lacking, prompting this review. This review aims to systematically examine and integrate existing evidence to offer a more complete understanding of the potential association between infertility treatments and stroke. The objective of this review is to determine the incidence of stroke/cerebrovascular accident among women who received ART and to compare the same with women who did not receive ART.

## Methods

### Study design and protocol registration

The systematic review was conducted as per Preferred Reporting Items for Systematic Reviews and Meta-Analyses (PRISMA) guidelines (Page *et al.* 2021). The study protocol was registered in the PROSPERO registry (ID: CRD42023491196).

### Eligibility criteria

The review included retrospective and prospective cohort studies conducted on women aged 15–55 years who experienced cerebrovascular accident/stroke with a history of undergoing different ART in the past. Studies on women who already had cardiovascular disease before undergoing ART treatments were excluded. In addition, we excluded case reports and case series from this review.

### Exposure and outcomes

All types of infertility treatments, such as *in vitro* fertilization (IVF), intrauterine insemination (IUI), follicular puncture and oocyte retrieval, intrauterine embryo transfer, and usage of drugs such as clomiphene citrate or a gonadotropin (follicle-stimulating hormone, human menopausal gonadotropin, human chorionic gonadotropin (HCG)), were considered as the exposure variables. The incidence of any stroke, including ischemic and hemorrhagic stroke, was the primary outcome of this study.

### Information sources

A systematic literature search was performed in PubMed, Embase, Cochrane, Web of Science (WoS), and CINAHL (Cumulated Index to Nursing and Allied Health Literature) through Nov 2023 on studies published in the English language in academic peer-reviewed journals. Two independent authors conducted the literature search using a structured search strategy.

### Search strategy

The search strategy employed to find relevant studies utilized MeSH terms such as infertility, subfertility, stroke, cerebrovascular accident (CVA), brain vascular accident, cerebrovascular stroke, cerebral stroke, acute cerebrovascular accident, and ischemic stroke (Supplement 1: detailed search strategy; see section on Supplementary materials given at the end of the article). Initially, keywords and their synonyms were searched using appropriate truncations, wildcards, and proximity searching techniques. Subject headings (MeSH for PubMed and Emtree for Embase) were also utilized for key concept searches across databases. The final search combined these results using Boolean operators. In addition, researchers in the field were contacted, and references of relevant articles were screened for further studies. No ethics approval or informed consent was required for this study, as it did not involve patient data collection. Gray literature and unpublished data were sought through a Google search.

### Study selection process

**Figure 1:**
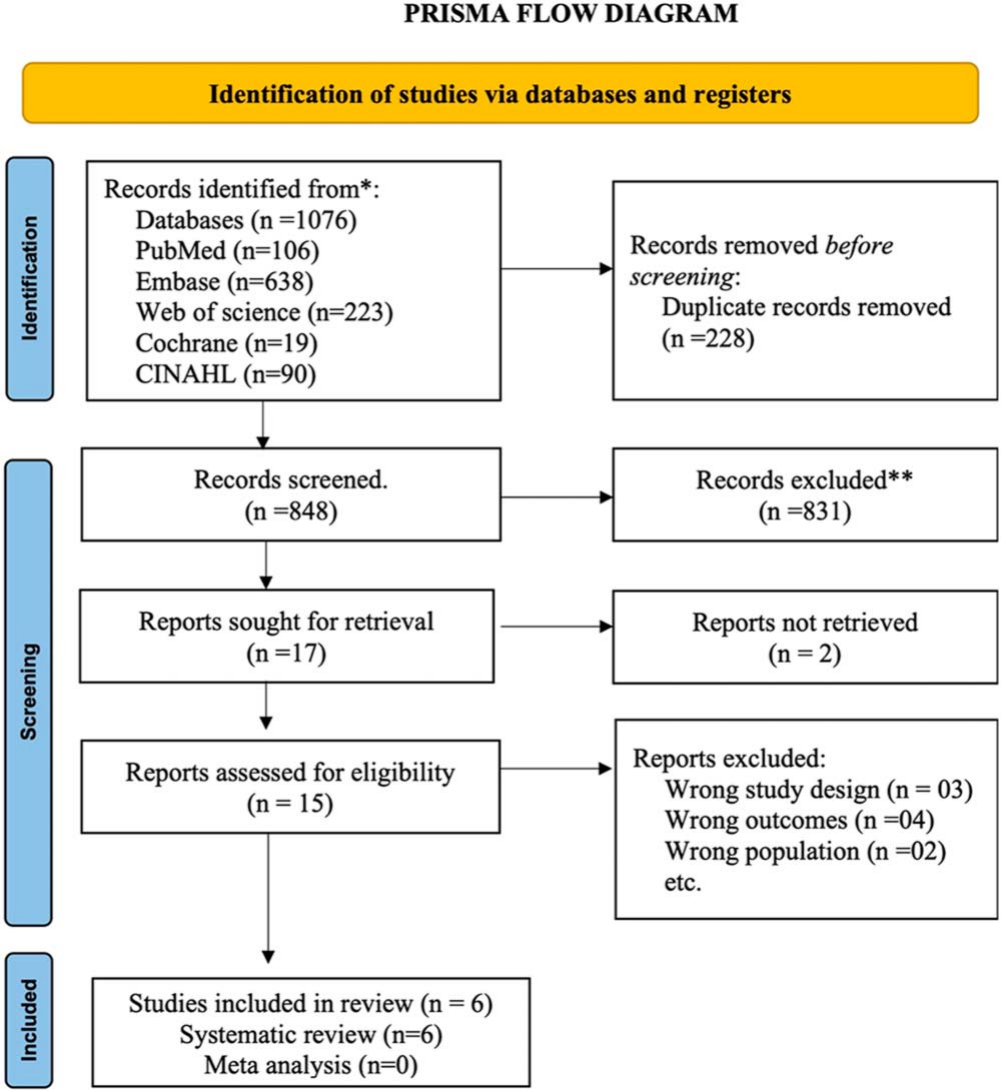
The preferred reporting items for systematic reviews and meta-analyses flowchart of the selection process of articles.

After de-duplicating, the studies were imported into the Rayyan.ai tool. In the first stage of screening, titles and abstracts were screened. Studies in line with the inclusion criteria were shortlisted and carried to the second stage of screening. In the second stage, all the shortlisted studies were screened for full text. The studies that were relevant to the objectives, fulfilling the inclusion criteria and outcome measures of interest, were taken ahead for data extraction. The studies that were relevant but did not satisfy a few aspects of eligibility criteria were labeled as ‘maybe’ or ‘conflict’ by the individual researcher. These contentious studies were later resolved by a third reviewer. Each stage involved two researchers (AAK and VD) who independently reviewed the level 1 and 2 screening and data extraction process, and a third reviewer settled disagreements regarding study eligibility (Fig. 1: PRISMA flow chart).

### Data collection process

All the articles were carefully screened, and data were extracted using the data extraction methodology used by the same researchers: demographics, research description (first author, publication year, aims, study design, and sample size), inclusion and exclusion criteria, outcomes, period of follow-up, type of ART received, duration or cycles of treatment for infertility, incidence of stroke, mortality due to stroke, and conclusions.

### Risk of bias in studies

The methodological quality of the included studies was evaluated in three domains: selection, comparability, and outcome/exposure. Assessment was independently conducted by two authors (AAK and VD) using the Newcastle–Ottawa Scale for cohort and case–control studies (Wells *et al.* 2000). In the comparability domain, age was the primary factor considered, with additional factors including hypertension, polycystic ovary syndrome (PCOS), body mass index, and income also evaluated. Regarding outcome assessment, a follow-up period of at least 5 years was deemed sufficient for stroke occurrences, given limited evidence on the ideal follow-up duration. Studies were scored on a maximum of nine points, categorizing study quality as high (score ≥8), moderate (score = 7), or low (score ≤6). If sufficient studies were available, funnel plots were utilized to explore reporting biases.

### Strategy for data synthesis

We assessed all studies for clinical and methodological heterogeneity with respect to population, intervention, outcomes, and study design. We intended to perform meta-analysis in case of the absence of clinical and methodological heterogeneity. As the included studies exhibited significant heterogeneity, we have presented the results qualitatively.

### Reporting bias assessment

Considering the small number of heterogeneous studies, we could not assess the reporting bias using a funnel plot or Egger test. In addition, the power was too low to distinguish chance from real asymmetry.

## Results

### Study selection

Out of 1,076 studies initially identified across all sources, 228 duplicates were removed, leaving 848 studies for initial screening on Rayyan.ai. These studies underwent level 1 screening based on their titles and abstracts, resulting in the exclusion of 831 studies that did not meet eligibility criteria. Seventeen studies progressed to level 2, or full-text, screening. Of these, two studies could not be accessed, and nine were subsequently excluded, three for incorrect study design, four for irrelevant outcomes, and two for incorrect study population. Finally, six studies, with a combined sample size of 406,438, were included in the qualitative synthesis and underwent data extraction (see Fig. 1).

### Study characteristics

Table 1 summarizes the characteristics of the included studies. Among the six studies, two were conducted in Canada (Udell *et al.* 2017, 2013), two in the USA (Murugappan *et al.* 2019, Sachdev *et al.* 2023), and one each in Sweden (Westerlund *et al.* 2014) and Taiwan (Ge *et al.* 2019). The publication years of the studies ranged from 2013 to 2023. Three studies were retrospective cohorts (Ge *et al.* 2019, Murugappan *et al.* 2019, Sachdev *et al.* 2023), and the remaining three were population-based cohorts (Udell *et al.* 2017, 2013, Westerlund *et al.* 2014). Participants’ mean age varied from 15 to 55 years, with sample sizes ranging from 4,710 to 287,813 and a pooled sample size of 406,438. Follow-up durations ranged from 6.2 months to 9.7 years. Exposure and outcome ascertainment methods included the Swedish IVF Register, Maternal and Birth Register, health insurance plan codes, and ICD 9, 10 codes. Exposures examined included infertility treatments such as IVF, IUI, follicular puncture, oocyte retrieval, intrauterine embryo transfer, and medications such as clomiphene citrate or gonadotropins (FSH, HMG, HCG). Outcomes examined included stroke, cerebrovascular events, cardiac ischemia, coronary heart disease, and CVD. Notably, Sachdev *et al.* (2023) reported separate incidence rates for ischemic and hemorrhagic stroke.

**Table 1:** Characteristics of included studies.

Reference	Country	Study design	Study population	Age, years	Sample size (SG) vs (CG)	FUY, median	Exposure ascertainment	Outcomes	Effect measure	Effect size, HR (95% CI)		Study quality
										Crude	Adjusted	
Sachdev *et al.* (2023)	USA	RCS	Women undergoing infertility treatment vs women with spontaneous conception	15–54	287,813 vs 31,052,178	6.2*^,^^†^	ICD 9,10; PCS coding system	Hospitalization for non-fatal stroke	HR	1.76 (1.25–2.48)	1.66 (1.17–2.35)	High/good
Murugappan *et al.* (2019)	USA	RCS	Infertile women vs women receiving routine gynecologic care	20–45	64,345 vs 3,128,345	3.8 ± 3.3	ICD 9,10; procedure code (CPT), claims for clomiphene citrate or gonadotropins	Incidence of CAD, CVD	HR	1.44 (1.21–1.71)	1.18 (0.99–1.4)	High/good
Udell *et al.* (2017)	Canada	PBCS	Fertility failure vs fertility success	≤50	19,093 vs 9,349	8.4	ICD 9,10	Acute stroke, TIA, HF, TE	RRR	1.32 (1.22–1.44)^‡^	1.25 (1.15–1.37)^‡^	High/good
Ge *et al.* (2019)	Taiwan	RCS	Infertility medications vs controls	NR	4,710 vs 18,840	NR	ICD 9	Occurrence of CVD, VTE, DVT, PE, IS	HR	0.85, (0.70–1.03)	0.82, (0.68–0.99)	Moderate
Westerlund *et al.* (2014)	Sweden	PBCS	Successful IVF women vs controls	19–47	23,498 vs 116,960	8.6 ± 4.6*	Swedish IVF register/MBR register	Stroke and CHD, HT, DM	HR	1.24 (0.71–2.28)	1.27 (0.97–1.58)	High/good
Udell *et al.* (2013)	Canada	PBCS	Fertility therapy present vs fertility therapy absent	15–55	6,979 vs 1,179,774	9.7	Billing codes of Ontario health insurance plan including multiple forms of assisted reproduction, ICD 9, 10	CAD, stroke, TIA, HF, TE, VTE, chronic HT, DM	HR	2.14 (1.02–4.50)	1.14 (0.54–2.44)	High/good

* Mean.

^†^ Months.

^‡^ Crude relative rate ratio and adjusted relative rate ratio.

RCS, retrospective cohort study; PBCS, population-based cohort study; DM, diabetes mellitus; FUY, follow-up years; HF, heart failure; TE, thromboembolism; SG, study group; CG, control group; ICD, International Classification of Diseases; HR, hazard ratio; RRR, relative rate ratio; CAD, coronary artery disease; CHD, coronary heart disease; CVD, cardiovascular disease; HT, hypertension; NR, not reported.

### Risk of bias in studies

Of the six included studies, five studies were of high quality, and remaining one study was of moderate quality as per the Newcastle-Ottawa Scale (Wells *et al.* 2000). The study by Ge *et al.* (2019) was classified as moderate quality since it did not mention the length of the follow-up period among the women who underwent infertility treatments.

### Incidence of stroke

Studies have documented varying stroke incidences, with Udell *et al.* (2013) reporting the lowest at 16.00 (9.80; 26.12) per 100,000 person-years (Udell *et al.* 2013), and Sachdev *et al.* reporting the highest at 437.01 (360.93; 529.13) per 100,000 person-years (Sachdev *et al.* 2023). Sachdev *et al.* compared stroke incidences between women who conceived through infertility treatment and those who conceived naturally. They found that the incidence rates of any stroke were 37 per 100,000 in the infertility treatment group versus 29 per 100,000 in the spontaneous conception group. For hemorrhagic stroke, the rates were 18 per 100,000 and 12 per 100,000, respectively. There was a difference reported in the incidence of ischemic stroke (19 per 100,000 population) between both groups (Sachdev *et al.* 2023). Ge *et al.* focused exclusively on ischemic stroke, reporting hazard ratios (HRs) without specifying incidence rates (Ge *et al.* 2019). Other studies have reported cerebrovascular and cardiovascular disease incidences alongside chronic conditions, including stroke (Westerlund *et al.* 2014, Murugappan *et al.* 2019).

### Association between infertility and stroke

Different studies have shown varying HRs for the association between infertility treatment and stroke. The study by Sachdev *et al.* reported a significant positive association between infertility treatment and stroke. The reported HR was as high as 1.66 (95% CI: 1.17–2.35) (Sachdev *et al.* 2023). In contrast, the study by Ge *et al.*, reported an HR of 0.82 (95% CI: 0.68–0.99), which indicates infertility treatments being slightly protective for the occurrence of stroke (Ge *et al.* 2019). Although the remaining studies also reported a positive association, their confidence intervals included a null value. Overall, substantial variability in the effect sizes reported across the studies exists.

## Discussion

The review aimed to address gaps in understanding ART and stroke, citing limited recent studies with inconclusive findings. Conflicting evidence was reported across the studies regarding the risk of stroke with ART. Only one study reported a significantly higher HR for stroke (Udell *et al.* 2013). Other studies showed marginally higher overall HRs, which were statistically insignificant (Westerlund *et al.* 2014, Ge *et al.* 2019).

A prior meta-analysis by Liang *et al.* also found inconsistent and inconclusive evidence (HR = 1.07, 95% CI: 0.87–1.32) (Liang *et al.* 2022), recommending further research on different treatments and etiologies. Interestingly, Ge *et al.*’s large cohort study reported contradictory findings, suggesting infertility medications might reduce ischemic stroke risk (adjusted HR = 0.82, 95% CI: 0.68–0.99) and cardiovascular disease (adjusted HR = 0.83, 95% CI: 0.74–0.94) compared to controls (Ge *et al.* 2019). Similarly, Udell *et al.*’s (2017) Canadian study found fewer stroke and cardiovascular disease cases with fertility treatments (Udell *et al.* 2017). This might be due to increased health awareness among those seeking treatments and managing conditions and lifestyle factors influencing stroke risk. However, none of the studies critically evaluated the plausible biological pathways or the complex interplay of a plethora of potential risk factors.

**Figure 2:**
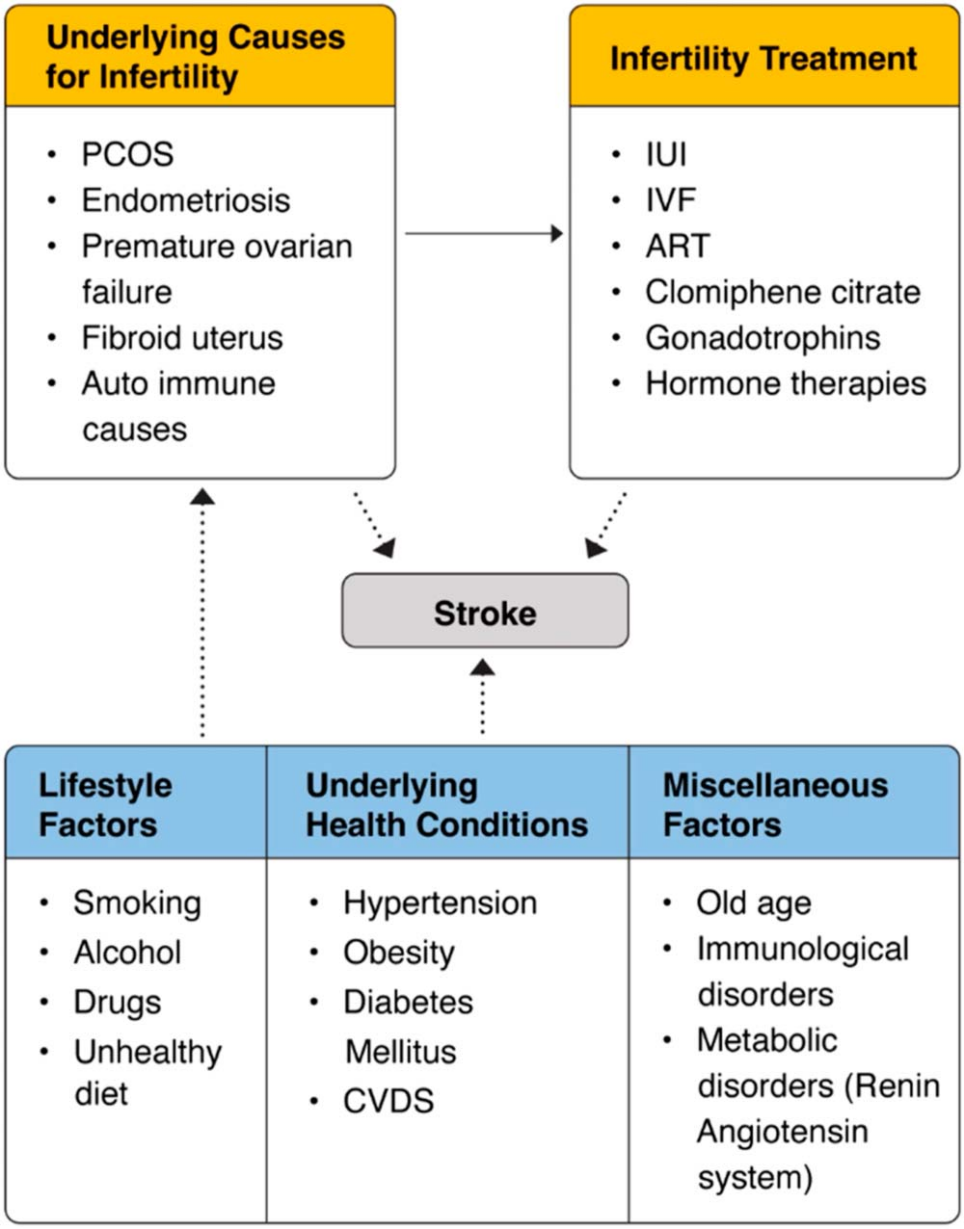
Conceptual framework of the hypothesized association of stroke among women who received infertility treatments.

Drawing such conclusions oversimplifies the complex, multifactorial relationships among infertility, its causes, treatments, psychological stressors related to infertility, and cardiovascular events or stroke (see Fig. 2). A comprehensive review of the existing literature unfolds valuable insights into the potential causes of stroke in women undergoing ART.

Background health conditions that increase the risk of infertility, the causes of infertility itself, and various medications and procedures in ART might contribute to a variable extent to the incidence of stroke in these women. Conditions such as PCOS, endometriosis, premature ovarian insufficiency, and impaired glucose tolerance might contribute to the underlying pathophysiology of stroke (Healy *et al.* 1994, Smith *et al.* 2021, Kicińska *et al.* 2023). The increased risk of hypercoagulability induced by gonadotropins, treatments with human chorionic gonadotropin (hCG), and gonadotropin-releasing hormone (GnRH), commonly used medications in infertility treatments (Gerotziafas *et al.* 2017). This hypercoagulability can lead to both arterial and venous thrombosis, posing a serious health risk. Similarly, ovarian hyperstimulation syndrome (OHSS), a common side effect of infertility treatments, is often associated with an increased risk of stroke (Aune *et al.* 1991, Kosteria *et al.* 2015). Furthermore, ART, even in the absence of OHSS, can trigger complications related to hypercoagulability and potential thromboembolic events (Dicpinigaitis *et al.* 2024). Women undergoing certain infertility treatments, such as IVF, could be linked to acute cardiovascular changes and serious blood flow issues in the placenta, potentially leading to preeclampsia, which may further increase the risk of hemorrhagic stroke (Miller & Leffert 2020, Magnus *et al.* 2023). The complex pathophysiology behind stroke in women of reproductive age group suggests multifaceted connections between infertility, ART, and stroke. Studies vary widely in terms of the populations, outcomes, follow-up durations, and assessment methods. These differences raise significant questions about the quality and consistency of available evidence. Diverse approaches to exposure and outcome assessment also question the reliability of combining results through meta-analysis.

### Heterogeneity in the study population

The systematic review included studies covering a wide age range. For instance, research by Sachdev *et al.* (2023) and Udell *et al.* (2017) encompassed participants as young as 15 years old, suggesting a low likelihood of fertility treatment in this age group. Conversely, studies also involved individuals up to 55 years old, where fertility treatment rates are likely higher, but potential links with stroke could be influenced by preexisting health conditions. The choice of the most relevant age group for such studies is debatable. Heterogeneity extended beyond age groups to include variations in study and comparator groups. Some studies compared women who conceived after infertility treatment with those who conceived spontaneously, while others compared outcomes between those who achieved fertility success and those who did not after treatment. The study population included in Murugappan *et al.* (2019) broadly defined the infertility cohort as women who were diagnosed, tested, or treated for infertility. Despite the wide-ranging inclusion criteria, the study was able to identify an association between cerebrovascular disease and the infertile group. However, subgroup analyses based on whether women were diagnosed, tested, or treated for infertility could help eliminate potential confounding in the observed associations. The diverse nature of the evidence precluded the pooling of data for meta-analysis, necessitating qualitative synthesis only.

### Heterogeneity in exposure assessment

Assessing exposure objectively and consistently is essential for scientifically valid conclusions on associations between risk factors and outcomes. However, this review showed significant variability in exposure assessment among studies. Three studies relied on insurance claims data, two defined exposure as any infertility treatment procedure, and one considered any infertility diagnosis as exposure (Udell *et al.* 2017, 2013, Westerlund *et al.* 2014, Ge *et al.* 2019, Murugappan *et al.* 2019, Sachdev *et al.* 2023). Furthermore, infertility treatment procedures were inconsistently categorized; for example, simple methods such as IUI were grouped with more complex ones like multiple IVF cycles. This lack of differentiation further extended to establishing dose-response relationships, such as comparing single versus multiple treatment cycles. This variability introduced substantial discrepancies in addressing the research questions and drawing conclusions across studies.

### Heterogeneity in outcome assessment

Similarly, the studies also varied in how they assessed outcomes. One study used a composite outcome including cardiovascular events, stroke, and other conditions, while others focused on CVD, hypertension, diabetes, and incident chronic diseases with cerebrovascular outcomes (Westerlund *et al.* 2014). Few studies specifically defined outcomes related to hospitalizations and stroke types (ischemic and hemorrhagic) (Sachdev *et al.* 2023). It is crucial to consider that chronic diseases may progress significantly before formal diagnosis, spanning preclinical and clinical stages (Megari 2013); however the likelihood of this occurring before cerebrovascular events is minimal.

### Conveniently ignoring potential confounders or underlying causes

The multifaceted pathogenesis involves numerous factors, including genetics, developmental origins, and environmental influences shaped by lifestyle (Fig. 2). Some studies in this review did not account for common risk factors such as obesity, smoking, physical inactivity, and underlying chronic diseases, such as diabetes or hypertension, whether undiagnosed or poorly controlled. This oversight could mask or exaggerate the true association. Adjusting for these variables would provide deeper insights and help isolate any potential specific impact of infertility treatments on stroke risk.

### Failure to address infertility vs infertility treatment procedures as a risk factor for stroke

Without establishing the underlying cause of infertility, it becomes challenging to attribute the increased risk of stroke solely to the treatment. Conditions such as PCOS and tubal defects, common causes of infertility, are closely related to hypertension and heart disease, complicating the association (Farland *et al.* 2023). In addition, few studies have reported the association between ovarian hyperstimulation syndrome (OHSS) and infertility treatments such as controlled ovarian stimulation (COS) or ovulation induction, which, in turn, can cause serious vascular problems (Namavar Jahromi *et al.* 2018, Koh *et al.* 2021). These treatments may increase stroke risk through factors such as estrogen-induced blood clotting, changes in blood clotting ability, or damage to blood vessels due to disruptions in the body’s hormonal balance (Dicpinigaitis *et al.* 2024). In contrast, a recent large-scale study observed a very low hospitalization rate of 4.06/100,000 population, with no ischemic strokes reported among them (Seitz *et al.* 2025). Given the current level of evidence, it is very challenging to attribute the risk of stroke to OHSS. Understanding these complexities is crucial for accurately assessing how infertility treatments affect stroke risk in women. However, many studies did not specify the duration of infertility treatment exposure. Some patients were advised to undergo multiple cycles, even after unsuccessful attempts, which could help establish a clearer dose-response relationship. Furthermore, some studies had insufficient follow-up periods to accurately determine stroke incidence.

The geographical distribution of infertility treatment and its link to stroke is uneven. Most studies included in our analysis focused on specific countries such as the USA, Canada, Sweden, and Taiwan. The global burden remains staggering, with limited evidence, highlighting a critical need for broader exploration to understand this immensely widespread issue.

### Strengths and limitations

Despite the limited number of studies, this review analyzed data from nearly 406,438 women. Our stringent inclusion criteria restricted the number of studies and data analyzed but effectively captured the associations of interest. Since data on infertility treatment and stroke were derived from medical records, recall bias is expected to be minimal. However, a few limitations should be acknowledged. The primary limitation is the scarcity of research data on both qualitative and quantitative aspects of infertility treatments and stroke. Due to the small and heterogeneous study pool, we could not assess reporting bias using funnel plots. In addition, insufficient studies precluded analysis by type of stroke or infertility treatment.

## Conclusion

In conclusion, the review could not conclusively establish the association between ART and stroke due to conflicting evidence reported across the included studies. It is still unclear whether the background conditions predisposing women to infertility, the direct causes of infertility, or ART itself increase the risk of stroke among women undergoing ART. It also remains unclear how long after ART the occurrence of stroke could be attributed to ART. Future research should focus on defining the timeframe for stroke risk after infertility treatments and identifying specific treatments linked to a higher risk. Robust prospective studies or real-world evidence investigations with suitable comparisons are essential to address these research gaps, thereby informing clinical practice and guiding patient care effectively.

### Recommendations

Based on our findings, it is imperative to emphasize that continuous monitoring of infertility treatments and long-term follow-up are crucial to detect and prevent stroke risks effectively. Addressing these knowledge gaps is vital for understanding the relationship between infertility treatments and stroke. We propose comprehensive stroke and cardiovascular risk assessments for women seeking infertility treatment, regardless of the type of treatment. Establishing longitudinal registries to monitor the occurrence of chronic diseases in women undergoing ART is also advised. These registries, with robust design and appropriate comparative groups to explore the complex interplay of factors associated with stroke and their underlying mechanisms, are critical to inform evidence-informed ART.

## Supplementary materials



## Declaration of interest

The authors declare that there is no conflict of interest that could be perceived as prejudicing the impartiality of the work reported.

## Funding

This research did not receive any specific grant from any funding agency in the public, commercial, or not-for-profit sector.

## Author contribution statement

AV and AKA conceptualized and designed the study and contributed to drafting and revising the manuscript. MMRG and AKA developed the systematic search strategy, screened studies, and extracted data. AV, AMA, VD, and PR conducted the quality assessment of included studies and contributed to the interpretation of findings. MMRG and AKA performed data analysis and assisted in result visualization. PR provided domain expertise, contributed to the discussion, and critically revised the manuscript. AV, MMRG, and AKA supervised the project, provided expert guidance, and finalized the manuscript for submission. All authors reviewed and approved the final version of the manuscript.
