# Supplementary material for: Association of infertility treatments and incidence of stroke among women: a systematic review
Source: Reprod Fertil. 2025 Oct 31;6(4):e240120. doi: 10.1530/RAF-24-0120 (PMC12583905; doi:10.1530/RAF-24-0120)
Supplement: Supplementary file 1 [file supplementary_materials.pdf]

Technical supplement; Comprehensive search strategy

PubMed search history: (dated: 14/11/2023)

Search number	Query	Results
1	"Infertility"[Mesh]	74,326
2	Infertility[Title/Abstract] OR "Subfertility"[Title/Abstract] OR "Sub-Fertility"[Title/Abstract]	70,609
3	#1 OR #2	110,239
4	"Stroke"[Mesh]	175,770
5	Stroke[Title/Abstract] OR "Cerebrovascular Accident"[Title/Abstract] OR "CVA"[Title/Abstract] OR "Brain Vascular Accident"[Title/Abstract] OR "Cerebrovascular Stroke"[Title/Abstract] OR "Cerebral Stroke"[Title/Abstract] OR "Acute Stroke"[Title/Abstract] OR "Acute Cerebrovascular Accident"[Title/Abstract] OR "Ischemic stroke"[Title/Abstract]	322,757
6	#4 OR #5	363,399
7	#3 AND #6	124
8	#3 AND #6	106

Embase search history: (Dated:14/11/23)

No.	Query	Results
#1	'infertility'/exp	154624
#2	infertility:ab,ti OR 'subfertility':ab,ti OR 'sub-fertility':ab,ti	99235
#3	#1 OR #2	180267
#4	'cerebrovascular accident'/exp	436724
#5	stroke:ab,ti OR 'cerebrovascular accident':ab,ti OR 'cva':ab,ti OR 'brain vascular accident':ab,ti OR 'cerebrovascular stroke':ab,ti OR 'cerebral stroke':ab,ti OR 'acute stroke':ab,ti OR 'acute cerebrovascular accident':ab,ti OR 'ischemic stroke':ab,ti	504704
#6	#4 OR #5	624736
#7	#3 AND #6	683
#8	#3 AND #6 AND [humans]/lim	638

Web of science search strategy: (Dated:14/11/23)

#	Search Query	Database	Results
1	ALL=(Infertility OR “Subfertility” OR “Sub-Fertility”)	Web of Science Core Collection	70872
2	ALL=(Stroke OR “Cerebrovascular Accident” OR “CVA” OR “Brain Vascular Accident” OR “Cerebrovascular Stroke” OR “Cerebral Stroke” OR “Acute Stroke” OR “Acute Cerebrovascular Accident” OR “Ischemic stroke”)	Web of Science Core Collection	622355
3	#1 AND #2	Web of Science Core Collection	223

Cochrane search strategy: (Dated:14/11/2023)

Date Run: 14/11/2023 19:29:46
Comment:

ID	Search	Hits
#1	MeSH descriptor: [Infertility] explode all trees	4494
#2	(Infertility OR “Subfertility” OR “Sub-Fertility”):ti,ab,kw	11027
#3	#1 OR #2	11201
#4	MeSH descriptor: [Stroke] explode all trees	15230
#5	(Stroke OR “Cerebrovascular Accident” OR “CVA” OR “Brain Vascular Accident” OR “Cerebrovascular Stroke” OR “Cerebral Stroke” OR “Acute Stroke” OR “Acute Cerebrovascular Accident” OR “Ischemic stroke”):ti,ab,kw	72330
#6	#4 OR #5	72942
#7	#3 AND #6	19

CINAHL search strategy: (Dated:14/11/23)

#	**Query**	**Results**
S1	TX Infertility OR “Subfertility” OR “Sub-Fertility”	23,142
S2	TX Stroke OR “Cerebrovascular Accident” OR “CVA” OR “Brain Vascular Accident” OR “Cerebrovascular Stroke” OR “Cerebral Stroke” OR “Acute Stroke” OR “Acute Cerebrovascular Accident” OR “Ischemic stroke”	165,878
S3	S1 AND S2	90
